# Primary cilia modulate balance of canonical and non-canonical Wnt signaling responses in the injured kidney

**DOI:** 10.1186/s13069-015-0024-y

**Published:** 2015-04-16

**Authors:** Shoji Saito, Björn Tampe, Gerhard A Müller, Michael Zeisberg

**Affiliations:** Department of Nephrology and Rheumatology, Göttingen University Medical Center, Georg August University, Robert-Koch Str. 40, 37075 Göttingen, Germany

**Keywords:** Cilia, Fibrosis, Epithelial

## Abstract

**Background:**

While kidney injury is associated with re-expression of numerous Wnt ligands and receptors, molecular mechanisms which underlie regulation of distinct Wnt signaling pathways and ensuing biological consequences remain incompletely understood. Primary cilia are increasingly being recognized as cellular ‘antennae’ which sense and transduce signals from the microenvironment, particularly through Wnt signaling. Here, we explored the role of cilia as modulators of canonical and non-canonical Wnt signaling activities involving tubular epithelial cells in the injured kidney.

**Results:**

We demonstrate that in the mouse model of unilateral ureter obstruction, progression of kidney injury correlates with increased expression of numerous Wnt ligands, and that increased expression of Wnt ligands corresponded with over-activation of canonical Wnt signaling. In contrast, non-canonical Wnt signaling dropped significantly during the course of kidney injury despite gradually increased expression of typical non-canonical and intermediate Wnt signaling ligands. We further demonstrate that in cultured tubular epithelial cells, cilia modulate balance between canonical and non-canonical signaling responses upon exposure to Wnt ligands.

**Conclusions:**

We provide evidence that in the context of renal injury, primary cilia act as molecular switches between canonical and non-canonical Wnt signaling activity, possibly determining between regenerative and pro-fibrotic effects of Wnt re-expression in the injured kidney.

**Electronic supplementary material:**

The online version of this article (doi:10.1186/s13069-015-0024-y) contains supplementary material, which is available to authorized users.

## Background

In principle, the kidney possesses a unique capacity to repair itself, even upon severe acute injury [1-3]. In clinical reality, however, complete regeneration of acute kidney injury is not always achieved [4,5]. Furthermore, impaired regeneration is a principal feature of fibrosis, a pathological scarring process which is the basis of chronic progressive kidney disease [6-8]. In clinical nephrology, both acute kidney injury and chronic progressive kidney disease pose major problems, as therapies to halt fibrosis or to induce regeneration of acute, or even chronic kidney injury, are not yet available [9]. Furthermore, the molecular mechanisms which discern between renal regeneration and chronic progressive fibrosis are still incompletely understood and insights derived from animal and cell culture studies have not yet been sufficiently translated into clinical context.

In this regard, Wnt signaling is being increasingly recognized as important mediator of both renal repair and progressive renal fibrosis. Wnt proteins (Wnt stands for Wingless-related integration site, a portmanteau of ‘int’ referring to the cancer-causing mammalian gene integration 1 and Wg referring to drosophila gene homolog known as wingless) in general are a family of secreted proteins, which act as ligands to activate distinct Wnt signaling pathways [10,11]. There are two principal Wnt-signaling pathways, the β-catenin-dependent ‘canonical’ Wnt pathway and the β-catenin-independent ‘non-canonical’ pathways [10]. In mammalians, there are currently 19 known Wnt ligand proteins and 10 different Frizzled-Wnt receptors, and since there is no clear ligand-receptor-pathway relationship, mechanisms that discern between activation of distinct canonical and non-canonical pathways are complex and only incompletely understood [10].

Wnt signaling in general plays multiple essential roles in organogenesis, carcinogenesis, and tissue homeostasis [11]. While in the kidney, Wnt-signaling is essential for nephron formation during kidney development, Wnt signaling activity is ominously silenced in the adult kidney [12]. Injury of the adult kidney is associated with re-expression of numerous Wnt ligands and receptors [13]. There are conflicting reports however, if such re-activation of Wnt signaling is beneficial and should be regarded as an attempt to re-activate developmental programs to repair the kidney [14-16] or if Wnt signaling is detrimental and contributes to progression of renal fibrogenesis [17-20].

While the role of Wnt signaling in the injured kidney is complex, majority of studies on canonical Wnt signaling demonstrate a detrimental role. A recent study implicated activity of β-catenin signaling - the prototypical read-out of canonical Wnt signaling - within tubular epithelial cells as a predictor of tubulointerstitial fibrosis and poor prognosis of kidney allografts [21]. Majority of studies on non-canonical Wnt signaling however report a beneficial pro-regenerative role. Because previous studies demonstrated a predominantly beneficial role of non-canonical Wnt signaling, yet a pro-fibrotic activity of canonical Wnt signaling, we hypothesized that the shift from non-canonical to canonical Wnt signaling may contribute to the switch between physiological repair and pathological fibrogenesis in response to Wnt re-expression, and we aimed to gain further insights into the mechanisms which modulate Wnt signaling responses in the injured kidney.

In this regard, primary cilia have emerged as principal modulators of Wnt signaling [22]. Primary cilia have been located on almost every mammalian cell type [23]. Primary cilia have emerged as cellular ‘antennae’ which sense and transduce signals from the microenvironment, particularly through Wnt signaling [24]. The kidney has emerged as the prime organ to study cilia biology as the most relevant genetic kidney diseases are caused by primary cilia defects (‘ciliopathies’). The role of cilia in kidney injury (in ‘nonciliopathies’) has not been studied in depth yet.

We observed that in the murine model of obstructive nephropathy irreversible tubulointerstitial fibrosis (7 days after ureter obstruction), co-incides with structural damage of primary cilia, overactivation of canonical Wnt signaling, and drop of non-canonical Wnt signaling activity. We provide causal evidence that loss of intact cilia contributes to the switch from non-canonical to canonical Wnt signaling in response to Wnt ligands by tubular epithelial cells, possibly contributing to the switch from reversible to irreversible kidney injury.

## Results

### Activation of Wnt signaling in kidney fibrosis

**Figure 1:**
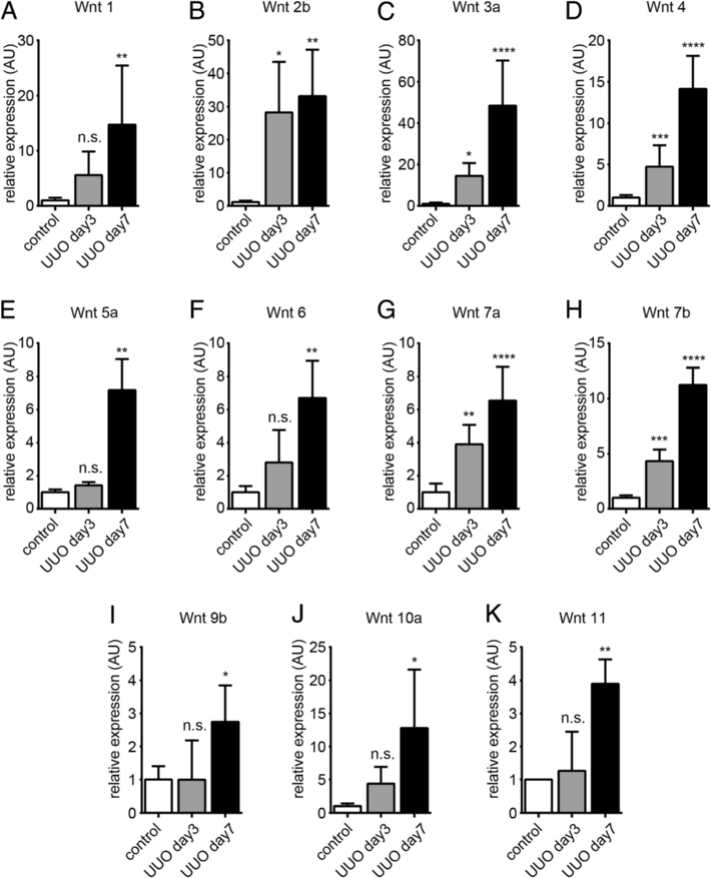
Renal expression of Wnt ligand genes upon unilateral ureter obstruction (**A-K**). We isolated total RNA from whole kidney tissues 3 and 7 days after ureter ligation and analyzed expression of putative Wnt ligand genes by quantitative real-time PCR. The bar graphs display relative mRNA expression of indicated Wnt ligand genes upon ureter ligation in relation to kidneys of sham-operated control mice. The arrows highlight robust expression of β-catenin in dilated tubules. Data is presented as means ± SD. *P < 0.05, **P < 0.01, ***P < 0.001, ****P < 0.0001, n.s. no significance, UUO, unilateral ureteral obstruction; *P* values were calculated respective to control.

To enable comparison of our study with previous reports regarding involvement of Wnt signaling and kidney disease, we first analyzed the dynamics of mRNA expression of Wnt ligand genes known to be expressed in adult kidneys in our mouse model of unilateral ureter obstruction (UUO) in C57/BL6 mice. Complete UUO initiates a rapid sequence of events in the obstructed kidney, leading within 24 h to reduce renal blood flow and glomerular filtration rate [25]. This is followed by hydronephrosis, tubular dilation, interstitial inflammatory infiltration (predominantly macrophages), and progressive interstitial fibrosis [25]. While kidney injury upon UUO is reversible when the ligation is successfully removed within 3 days, it is no longer spontaneously reversible after 7 days [26]. Quantitative real-time-polymerase chain reaction (RT-PCR) analysis of control kidneys (sham surgery), of kidneys 3 days after ureter ligation (initiating injury, potentially reversible) and 7 days after UUO (irreversible tubulointerstitial fibrosis) revealed that expression of Wnt2b, Wnt3a, Wnt4, Wnt7a, and Wnt7b were significantly elevated after 3 days of ureter ligation, after 7 days all tested Wnt genes (Wnt1, Wnt2b, Wnt3a, Wnt4, Wnt5a, Wnt6, Wnt7a, Wnt7b, Wnt9b, Wnt10a, and Wnt11) were markedly increased (Figure 1A,B,C,D,E,F,G,H,I,J,K).

**Figure 2:**
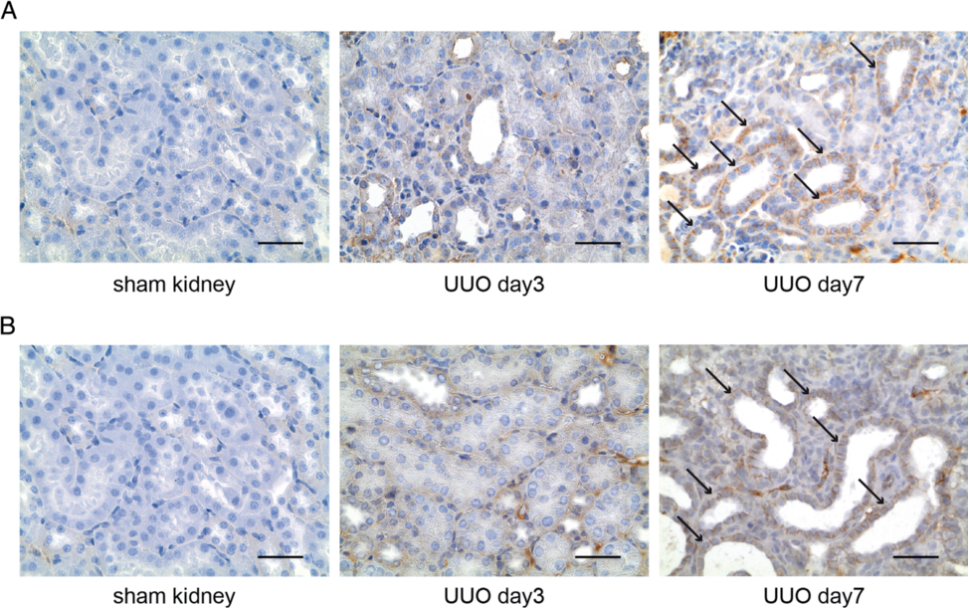
β-catenin immunolocalization in experimental obstructive nephropathy. We labeled kidneys of sham-operated mice and of mice which had been challenged with UUO with antibodies specific to (**A**) total β-catenin or with antibodies specific for (**B**) non-phosphorylated β-catenin (indicative of active canonical signaling). The pictures display representative photomicrographs of each groups, positive immunostaining is indicated by brown precipitate. The arrows highlight robust expression of β-catenin in dilated tubules. Scale bars: 100 μm. UUO, unilateral ureteral obstruction.

We next performed immunostaining experiments using antibodies to β-catenin, the principal mediator of canonical Wnt signaling (which had been implicated as marker of poor prognosis in chronic allograft nephropathy [27-29]). While in control kidneys, β-catenin protein was barely detectable (which is in line with several previous studies [13]), only few interstitial cells were β-catenin-positive after 3 days of ligation, whereas after 7 days robust β-catenin immunolabeling was observed after 7 days of ligation within tubular epithelial cells, most prominently in dilated tubules (Figure 2A). Similar immunolocalization was observed when antibodies to non-phosphorylated β-catenin were used, indicative of highly active canonical Wnt signaling (referred to as canonical Wnt signaling over-activation) (Figure 2B) [30]. In summary, we observed that chronic kidney injury upon ureter ligation was associated with robust increased of Wnt ligand expression and subsequent over-activation of canonical Wnt signaling, specifically in dilated tubules.

**Figure 3:**
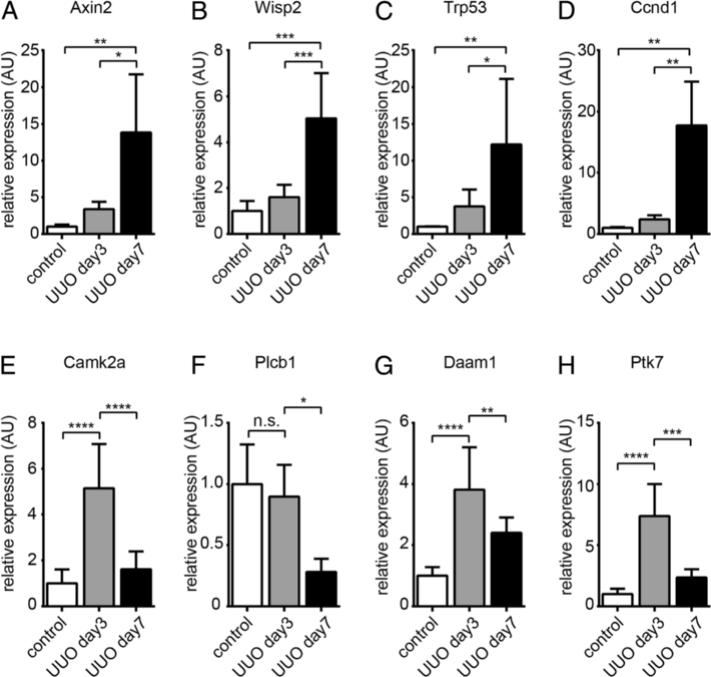
Relative expression of canonical and non-canonical Wnt target genes upon unilateral ureter obstruction. We isolated total RNA from whole kidneys of sham-operated control mice and of mice which had been challenged with UUO and analyzed expression of target genes of canonical Wnt signaling (Axin2, Wisp2, Trp53, and Ccnd1, graphs **A**-**D**) and of non-canonical Wnt target genes (CamK2a, Plcb1, Daam1, and Ptk7, graphs **E**-**H**). Data is presented as means ± SD. *P < 0.05, **P < 0.01, ***P < 0.001, ****P < 0.0001, n.s. no significance; UUO, unilateral ureteral obstruction.

To further substantiate our findings of unequivocal increase of Wnt ligand expression and Wnt signaling activity, we next analyzed the expression of several Wnt target genes as indicators of canonical and non-canonical Wnt signaling activity [31,32]. Corresponding with increase of Wnt ligand expression and β-catenin immunostaining, expression of all target genes of canonical Wnt signaling tested, namely Axin2, Wisp2 (Wnt1 inducible signaling pathway protein 2), Trp53 (tumor protein p53), and Ccnd1 (cyclin D1), were substantially increased 7 days after of ureter ligation (Figure 3A,B,C,D) but not yet after 3 days, correlating with dynamics of Wnt ligand gene expression (highest after 7 days of UUO) and β-catenin immunostaining (prominent after 7 days of UUO). Expression of target genes of non-canonical Wnt signaling however displayed a different pattern (Figure 3E,F,G,H). Expression levels of calcium/calmodulin-dependent protein kinase type II alpha chain (Camk2a), disheveled-associated activator of morphogenesis 1 (Daam1), and tyrosin protein kinase-like 7 (Ptk7) were significantly increased after 3 days of UUO, and expression of all non-canonical target genes tested dropped substantially from days 3 to 7. In summary, while canonical Wnt signaling correlated with increased Wnt ligand expression and progressive fibrosis, non-canonical Wnt signaling followed a distinct pattern, as target gene expression peaked after 3 days of ureter ligation but dropped significantly until day 7 with emerging tubulointerstitial fibrosis. While the peaking of non-canonical Wnt signaling could be explained with increased expression of Wnt ligand genes Wnt2b, Wnt3a, Wnt4, Wnt7a, and Wnt7b (all typical inducers of non-canonical Wnt signaling), drop of non-canonical Wnt signaling activity after 7 days was unexpected, because expression of all Wnt ligand genes was further increased, suggesting that additional modulatory mechanisms were at play.

### Correlation of Wnt signaling switch and impairment of cilia

**Figure 4:**
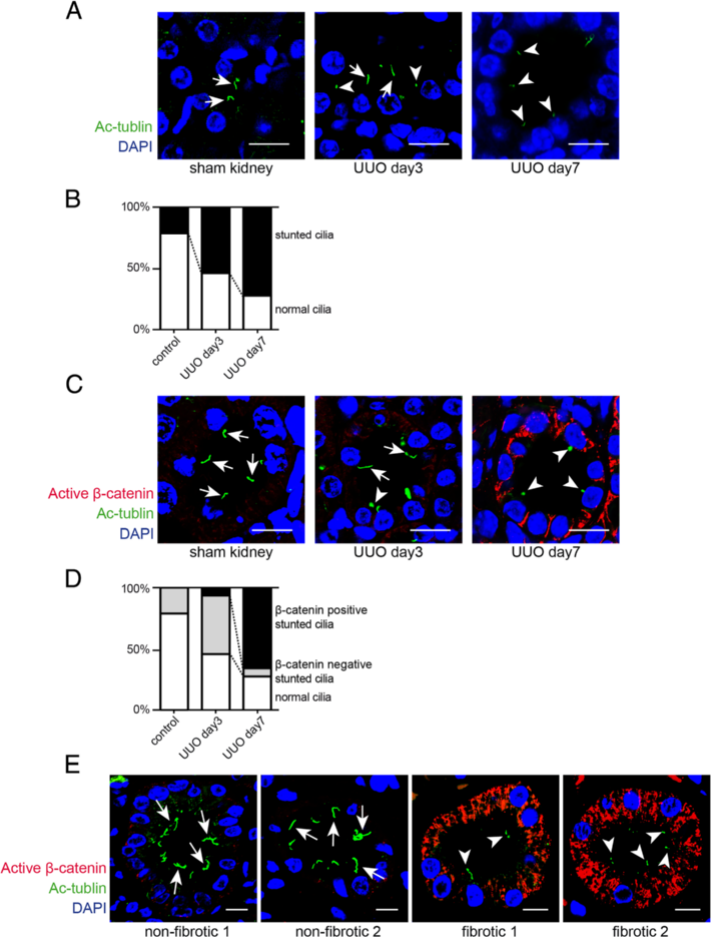
Stunted cilia and canonical Wnt signaling in experimental obstructive nephropathy. (**A**) Paraffin-embedded kidney sections were labeled with antibodies to acetylated α-tubulin (Ac-α-tubulin) (green). Nuclei were labeled with DAPI (blue). Staining was analyzed using a confocal microscope. The pictures display representative confocal photomicrographs of labeled sections of sham-operated mice (left) and of kidneys 3 days (middle) and 7 days (right) after ureter obstruction. Scale bars: 10 μm. Arrows indicate normal cilia and arrowheads indicate stunted cilia. (**B**) The graph summarizes the relative distribution of normal and stunted cilia in each group. (**C**) Immunofluorescence double-labeling experiments were performed using specific antibodies to acetylated α-tubulin (cilia) and non-phosphorylated β-catenin (active canonical Wnt signaling). Nuclei were stained with DAPI. The pictures display representative photomicrographs of sections from sham-operated control mice (left) and post-obstructive (days 3 and 7) kidneys. Non-phosphorylated β-catenin immunostaining was most abundant in tubules with stunted cilia. Arrows indicate normal cilia and arrowheads display stunted cilia. Scale bars: 10 μm. (**D**) The graph shows relative distribution of normal cilia and stunted cilia regarding to β-catenin expression in each group. (**E**) Non-fibrotic and fibrotic kidney sections from human biopsies were labeled with acetylated α-tubulin (cilia) and non-phosphorylated β-catenin. Pictures display representative confocal photomicrographs of each sample. Arrows indicate normal cilia and arrowheads indicate stunted cilia. Scale bars: 10 μm. UUO, unilateral ureteral obstruction; DAPI, 4′,6-diamidino-2-phenylindole.

Because our data had suggested reciprocal expression patterns of canonical and non-canonical Wnt signaling target genes - despite unequivocally increased expression of Wnt ligand mRNAs - we next hypothesized that an additional regulatory element is involved in determining between canonical and non-canonical Wnt signaling in context of kidney fibrosis. In this regard, primary cilia (here referred to as cilia) have emerged as important determinant of Wnt signaling [33]. Cilia have been located on almost every mammalian cell type [23]. Primary cilia are increasingly being recognized as cellular ‘antennae’ which sense and transduce signals from the microenvironment, particularly through Wnt and Sonic hedgehog signaling, and based on previous studies in *Xenopus* and zebrafish, it has been speculated that cilia can function as molecular switches between canonical and non-canonical signaling pathways [34,35]. To gain insights into the possible involvement of cilia in regulation of Wnt signaling in the UUO model, we analyzed primary cilia on tubular epithelial cells by immunolabeling using antibodies specific for acetylated α-tubulin followed by confocal microscopy. After 7 days of ureter ligation, cilia on tubular epithelial cells appeared stunted and cilia density decreased, while cilia were still unaltered after 3 days of ureter ligation (Figure 4A,B) [36,37]. We next performed immuno-double-labeling experiments using antibodies to both acetylated α-tubulin and β-catenin revealed and quantified normal ciliated cells (never β-catenin positive); cells with stunted cilia which stained negative for active β-catenin and cells with stunted cilia which stained positive for active β-catenin in the kidneys of mice without, 3 days after UUO and 7 days after UUO, respectively. As compared to control kidneys, tubular cells with stunted cilia became abundant after 3 days of UUO, albeit most of these cells were β-catenin negative. After 7 days of UUO, cells with stunted cilia in which β-catenin signaling was active became the most frequent cellular phenotype (Figure 4C,D). To gain insights into a possible correlation of impairment of cilia and canonical Wnt signaling responses in progressive kidney fibrosis in patients, we performed immune-double-labeling experiments using antibodies to α-tubulin and β-catenin on kidney biopsy specimen which had been scored by a pathologist either as non-fibrotic or fibrotic (>50% and >90%, respectively) (Additional file 1). While in non-fibrotic kidneys β-catenin immunolabeling was not detectable correlating with abundant presence of normal appearing cilia, cystic dilated tubules within fibrotic kidneys displayed substantial cytoplasmatic β-catenin labeling, correlating with stunted or lost cilia (Figure 4E). Of note, observed β-catenin immunolabeling observed in human kidney biopsies was far more robust than that observed in mice, mirroring previous reports [21].

In summary, our studies revealed that progression of kidney injury upon ureter ligation is associated with increased canonical and decreased Wnt signaling and that such Wnt signaling switch is associated with increased Wnt ligand expression and progressive loss of cilia.

**Figure 5:**
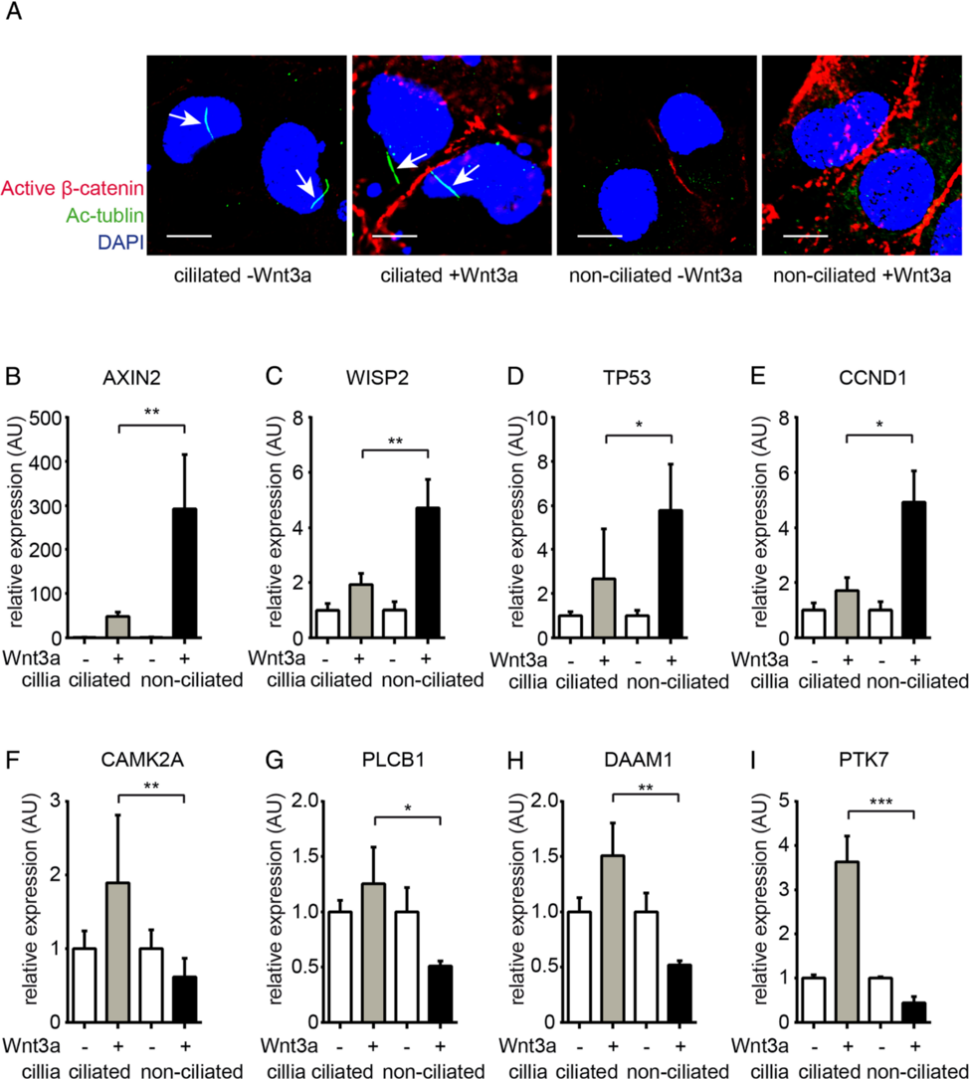
Reciprocal responses against Wnt in ciliated and non-ciliated cells. We compared induction of canonical and non-canonical Wnt target genes in response to Wnt3a in cultured ciliated and non-ciliated HK2 tubular epithelial cells. The graphs display relative mRNA expression of indicated genes in ciliated versus non-ciliated cells with or without Wnt3a in culture media. (**A**) Ciliated and non-ciliated HK2 cells were methanol fixed and were labeled with antibodies against acetylated α-tubulin (cilia) and non-phosphorylated β-catenin. Higher expression of active β-catenin was observed in Wnt3a-treated non-ciliated cells. Arrows highlight normal cilia on epithelial cell. Scale bars: 10 μm. (**B**-**E**) Expression of canonical Wnt signaling target genes (AXIN2, WISP2, TP53, CCND1) was substantially induced in ciliated cells but not in non-ciliated cells. (**F**-**I**) Addition of Wnt3a to culture media induced expression of non-canonical target genes in ciliated cells but not in non-ciliated HK2 cells (CAMK2A, PLCB1, DAAM1, PTK7). Data is presented as means ± SD. *P < 0.05, **P < 0.01, ***P < 0.001, n.s. no significance, DAPI, 4′,6-diamidino-2-phenylindole. *P* values were calculated respective to each sample without treatment.

To gain further insights into the modulatory role of cilia on canonical and non-canonical signaling in response to Wnt ligands, we next utilized a cell culture model of ciliated and non-ciliated HK2 human proximal tubular epithelial cells (Figure 5A) [38]. We exposed ciliated and non-ciliated HK2 cells to Wnt3a (which is known to elicit both canonical and non-canonical signaling responses and which we found to be substantially upregulated upon UUO) and analyzed mRNA expression of putative canonical and non-canonical Wnt signaling target genes. We observed that in non-ciliated HK2 cells increase of β-catenin activation and expression of canonical Wnt target genes (AXIN2, WISP2, TP53, and CCND1) was further augmented (Figure 5B,C,D,E) as compared to ciliated cells. In contrast, expression of non-canonical Wnt signaling target genes decreased significantly when Wnt3a was added to cell culture media of non-ciliated cells but increased when cells were ciliated (Figure 5F,G,H,I).

## Discussion

Here, we report two novel findings which add new aspects and possible explanation to the complexity of Wnt signaling in the context of kidney disease. While our study is in line with previous reports of increased canonical Wnt signaling during renal fibrogenesis (an intuitive finding in view of robust overexpression of multiple Wnt ligand genes), we report for the first time a distinct pattern of non-canonical Wnt signaling activity, as expression of non-canonical Wnt target genes drops during disease progression (despite increased expression of Wnt ligand genes). Second, we provide evidence for the first time that primary cilia (which deteriorate during progression of tubulointerstitial fibrosis) play a causal role in discerning between canonical and non-canonical Wnt signaling pathways, likely impacting the net-effect of Wnt activity in the injured kidney.

While several previous studies agree that kidney injury is associated with increased expression of Wnt ligands [13], their impact on disease progression has been controversial as some studies concluded that Wnt signaling contributes causally to progression of kidney fibrosis [17-20] whereas others reported that Wnt signaling is beneficial for the injured kidney [14-16]. However, most of these prior conflicting studies focused on canonical Wnt signaling, whereas the role of non-canonical Wnt signaling has been largely overlooked. Several studies reported however, that non-canonical Wnt signaling contributes causally to renal repair upon acute kidney injury, and hence, it is attractive to speculate that the sum effect of Wnt signaling in the injured kidney is at least in part determined by cilia-dependent non-canonical Wnt signaling. Context-dependent changes in the sum effect of Wnt signaling due to a switch from predominantly non-canonical to canonical Wnt signaling is not without precedent [39], and we believe that the intensity of non-canonical Wnt signaling is not the only determining factor in the context of kidney disease either [40]. Such thinking may be further supported by our observation that cilia impairment and impaired non-canonical Wnt signaling canonical over-activation are only observed after 7 days of ureter ligation (when kidney injury is irreversible) but not yet after 3 days (when kidney injury is still reversible when the ligature is removed) [41].

With regard to conflicting interpretations of the sum effect of Wnt signaling on kidney fate, it may be also important to note that loss of cilia appears to be model specific and that we did not observe cilia stunting or β-catenin immune-labeling in other murine models of kidney disease (data not shown). Similarly, impact of genetic background (in our case C57BL/6 mice were used) remains unclear.

We are aware of the fact that the strongest clinical evidence for a detrimental role of canonical Wnt signaling stems from a study which analyzed utility of tubular β-catenin immunostaining as predictor of chronic allograft nephropathy [27]. Of note, in this study, tubular β-catenin immunostaining was not analyzed as a read-out for canonical Wnt-signaling overactivation but as biomarker for epithelial-mesenchymal transition (EMT) [27]. In the context of kidney disease, EMT (defined as acquisition of mesenchymal properties and loss of epithelia functions through induction of specific EMT-transcriptional programs) is considered to contribute causally to progression of tubulointerstitial fibrosis through impairment of tubular functions and also through contribution to interstitial fibroblast accumulation [6,42]. In this regard, Wnt-initiated β-catenin signaling was identified as original EMT pathway in palate formation [43,44], and numerous studies corroborated β-catenin signaling as the master regulator of EMT in the context of embryonic development, cancer progression, and organ fibrosis [45-47]. Furthermore, previous studies linked Wnt-induced EMT to p53 accumulation, and our studies demonstrate increased p53 mRNA expression in response to cilia impairment [48,49]. Hence, one may speculate that loss of cilia in injured tubular epithelial cells contributes to progression of chronic kidney disease through facilitating a switch from non-canonical to canonical Wnt signaling, ultimately contributing to EMT of affected epithelial cells.

Because cilia play a central role in pathogenesis of hereditary cystic kidney diseases (because most mutations causing hereditary cystic kidney diseases affect genes which encode for cilia proteins), it is tempting to compare our observations with existing polycystic kidney disease (PKD) literature. In this regard, increased Wnt signaling *per se* is considered to contribute causally to manifestation of autosomal dominant polycystic kidney disease [50]. Cystogenesis associated with nephronophthisis (NPH), which is caused by mutations of genes encoding for nephrocystin proteins (NPHPs) is considered to be driven by over-activation of canonical Wnt signaling, and NPHP4 causally inhibits canonical Wnt signaling through enabling proteolytic degradation of β-catenin [51]. In this context, it is attractive to speculate that observed cilia loss and β-catenin over-activation in cystically dilated tubules in the UUO model contribute to progression of tubular atrophy through mechanisms which are similar to those which contribute to cystogenesis in PKD. While such studies are beyond the scope of this study, qualitative analysis of stunted cilia in the UUO model may provide important insights into the role of cilia in modulation of Wnt signaling and progression of chronic kidney disease in the future.

We are aware that our study focuses entirely on the impact of cilia and Wnt signaling in tubular epithelial cells. If such mechanism is relevant for other cell types such as fibroblasts remains to be seen.

## Conclusions

Primary cilia favor non-canonical Wnt signaling responses in tubular epithelial cells, while loss of intact cilia augments canonical Wnt signaling in response to Wnt ligands. As β-catenin superactivation has been identified as biomarker for negative outcome of chronic kidney disease and kidney injury is associated with increased expression of most Wnt ligands, impairment of primary cilia likely contributes causally to renal fibrogenesis by serving as molecular switch from non-canonical to canonical Wnt signaling responses.

## Methods

### Unilateral ureteral obstruction

Eight- to twelve-week-old *CD57BL/6* mice were anesthetized with isoflurane inhalation. Analgesia was performed by subcutaneous Buprenorphine injection. The ureter was separated from the surrounding tissues, and two ligatures were placed about 5 mm apart in upper two thirds of the ureter of the left kidney to obtain reliable obstruction. Mice were sacrificed at indicated time points after ureter ligation [52]. All studies had been carried out with the approval of local authorities (Tierschutzkommission Universitätsmedizin Göttingen) and LAVES (Landesamtes für Verbraucherschutz und Lebensmittelsicherheit; G 12/685).

### Immunohistochemistry

Fomalin-fixed, paraffin-embedded kidney sections from mice and human biopsy samples were deparaffinized in xylene and dehydrated through graded alcohols. Antigen retrieval was undertaken in a steam cooker for 40 min in Target retrieval solution (Dako, Glostrup, Denmark). Endogenous peroxidase was blocked with 3% hydrogen peroxide (H_2_O_2_) in methanol for 30 min. Samples were blocked with 2.5% horse serum (Dako, Glostrup, Denmark). After the blocking step, sections were incubated with primary antibody for overnight at 4°C in a humidified chamber, and the biotinylated secondary antibody (Dako, Glostrup, Denmark) was applied for 30 min at room temperature. The reaction products were visualized using 3,3′-diaminobenzidinetetrahydrochloride (DAB) (Dako, Glostrup, Denmark) and slides were counterstaining with hematoxylin. Mouse monoclonal β-catenin antibody (sc-7963: Santa Cruz Biotechnology Inc., Santa Cruz, CA, USA) and rabbit monoclonal non-phospho (Active) β-catenin antibody (#8814: Cell Signaling, Danvers, MA, USA) were used as primary antibodies.

### Immunofluorescence staining

Immunofluorescence staining of cells was performed in chambered slides (BD Bioscience, San Jose, CA, USA). Cells were seeded in chambered slides and fixed in ice-cold methanol for 10 min. Slides were then washed in phosphate-buffered saline (PBS) three times, and they were incubated with blocking solution (3% BSA in PBS) for 30 min. For immunofluorescence staining of mouse tissues, formalin-fixed, paraffin-embedded sections were used. Slides were deparaffinized and dehydrated. Mouse monoclonal acetylated α-tubulin antibody (Sigma-Aldrich, St. Louis, MO, USA) and non-phospho β-catenin antibody (Cell signaling, Danvers, MA, USA) was used as primary antibodies. Samples were then stained with Alexa Fluor 488-conjugated anti-mouse IgG and Alexa Fluor 568-conjugated anti-rabbit IgG antibody (Life technologies, Carlsbad, CA, USA) and mounted with VECTASHIELD Mounting Medium (Vector laboratories, Burlingame, CA, USA) with DAPI (4′,6-diamidino-2-phenylindole).

### Confocal microscopy and image analysis

Cells were viewed by using the Axiovert-200 inverted light and epifluorescence microscope (Carl Zeiss, Oberkochen, Germany) or using the confocal LSM 780 microscope (Carl Zeiss, Oberkochen, Germany). Images of cilia were captured from randomly chosen high-power fields (40× objective), when the full extent of cilia could be visualized in a single plane of focus. Cilia were scored as either possessing a full length primary cilia (>2 μm) or possessing a stunted primary cilia (<2 μm). Cilia from ten proximal and ten distal tubules were measured for each mouse.

### Cell culture

HK2 human proximal tubular epithelial cells were cultured in DMEM (Gibco, Carlsbad, CA, USA) supplemented with 100 g/mL penicillin, 100 g/mL streptomycin, and 10% fetal bovine serum (FBS, Cellgro, Manassas, VA, USA) at 37°C in 5% CO_2_. For immunostaining of cilia in culture, cells are grown as a monolayer on chambered slides (BD Bioscience, San Jose, CA, USA) and for RNA isolation in six-well plates (Greiner Bio-One, Kremsmunster, Austria). To generate ciliated and non-ciliated tubular epithelial cells, we seeded HK2 cells at different densities (six-well plates; 5,000 *vs* 30,000 per well in six-well plates) and starved cells for 48 h without serum to induce cell cycle exit and ciliogenesis as described previously [38]. Upon serum starvation, we exchanged media with growth factor-free control media or with media supplemented with 100 ng/ml recombinant Wnt3a (R&D systems, Minneapolis, MN, USA) for 48 h.

### RNA isolation

Tissue and cells were dissolved in TRIZOL (Invitrogen, Carlsbad, CA, USA), and tissue were shredded using TissueLyser LT (Qiagen, Hilden, Germany). RNA was isolated by using PureLink RNA Mini Kit (Ambion, Carlsbad, CA, USA) according to the manufacture’s protocol.

### Quantative real-time PCR quantification

For SYBR-based real-time PCR, cDNA synthesis was performed by using DNase I (Sigma-Aldrich, St. Louis, MO, USA) treatment and SuperScript II Reverse Transcriptase (Invitrogen, Carlsbad, CA, USA) according to the manufacturer’s protocol. Reverse-transcripted cDNA was added to the reaction mixture containing the primer pair and diluted with Fast SYBR Green Master Mix (Applied Biosystems, Carlsbad, CA, USA). PCR reactions were performed in a 96-well reaction plate using the StepOne Real-Time System (Applied Biosystems, Carlsbad, CA, USA) and were done in triplicates. All qRT-PCR data for RNA expression analysis were calculated using the ΔΔCT method. For statistical evaluation, *t*-test or a one-way ANOVA was used. Oligonucleotide sequences are shown in Additional file 2.

### Kidney biopsies

The use of parts of kidney biopsies for research purposes was approved by the Ethics Committee of the University Medical Center Göttingen, and written consent was obtained from all subjects before kidney biopsy. Detailed clinical patient data are presented in Additional file 1.

## Additional files

Additional file 1:
**Patient data.** Patient information of kidney biopsies. Additional file 2:
**Primer information.** Primer sequences used for qRT-PCR.

## Abbreviations

BSA: Bovine serum albumin
Camk2a: Calcium/calmodulin-dependent protein kinase type II alpha chain
Ccnd1: Cyclin D1
Daam1: Disheveled-associated activator of morphogenesis 1
DAB: 3,3′-diaminobenzidinetetrahydrochloride
DAPI: 4′,6-diamidino-2-phenylindole
EMT: Epithelial-mesenchymal transition
GAPDH: Glyceraldehyde 3-phosphate dehydrogenase
LAVES: Landesamtes für Verbraucherschutz und Lebensmittelsicherheit
NPH: Nephronophthisis
NPHP: Nephrocystin protein
PBS: Phosphate-buffered saline
PKD: Polycystic kidney disease
Ptk7: Tyrosin protein kinase-like 7
RT-PCR: Real-time-polymerase chain reaction
TP53: Tumor protein p53
Trp53: Tumor protein p53
UUO: Unilateral ureteral obstruction
Wisp2: Wnt1 inducible signaling pathway protein 2
