# Supplementary material for: Primary cilia modulate balance of canonical and non-canonical Wnt signaling responses in the injured kidney
Source: Fibrogenesis Tissue Repair. 2015 Apr 16;8:6. doi: 10.1186/s13069-015-0024-y (PMC4404279; doi:10.1186/s13069-015-0024-y)
Supplement: Additional file 2: — Primer information. Primer sequences used for qRT-PCR. [file 13069_2015_24_MOESM2_ESM.pdf]

Oigonucleotides for qRT-PCR

Target gene	Forward primer sequence<br><br>Reverse primer sequence	Supplier
mWnt1	GCCTCGGAGCCATTGAACA<br>GCAAATTATTTACACAGTGATGAGGA	Primerdesign, UK
mWnt2b	GGGGATGTTGTCACAGATCA<br>CTGCTGCTGCTACTCCTGACT	
mWnt3a	TGCCATGAACCGTCACAAC<br>CCAGCAGGTCTTCACTTCAC	Primerdesign, UK
mWnt4	CTGGAGAAGTGTGGCTGTGA<br>GGACTGTGAGAAGGCTACGC	
mWnt5a	TTACACAACAATGAAGCAGG<br>ACACTCCATGACACTTACAG	
mWnt6	GGTTCGAGAATGTCAGTTCC<br>ATTGCAAACACGAAAGCTG	
mWnt7a	CTAGCTCTCGGAACTGTGGC<br>TTACACAATAACGAGGCGGG	
mWnt7b	CCAGGCCAGGAATCTTGTT<br>ACGTGTTTCTCTGCTTTGGC	
mWnt9b	CGAGGAGATGCGAGAGTGC<br>GGAAGGGTGTCAGGACCTC	
mWnt10a	AGATCTGATTGACATTCCTCC<br>TGAGCTAGGAACAGAAAGAG	
mWnt11	TGAAGGACTCAGAACTTGTG<br>GGAAGTCTTGTTGCACTG	
mAxin2	ACGATTCCATGTCCATGAC<br>AATGAGGTAGAGACACTTGG	Primerdesign, UK
mWisp2	CTTCTTGGACTTCTATGTGCTTGT<br>CGGGCAGGGTGTTCTCAG	Primerdesign, UK
mTrp53	GAACCGCCGACCTATCCTTA<br>GCACAAACACGAACCTCAAA	Primerdesign, UK
mCcnd1	AACCATTCCATTTCAAAGCACTT<br>ATCCCCATCCATTCCATTAGAAC	Primerdesign, UK
hAXIN2	TGTGGGCAGTAAGAAACAG<br>CTCGGGAAATGAGGTAGAG	Primerdesign, UK
hWISP2	CGGAACATAAAGACTCACAGGT<br>AGGCAGAGGAGGGAGAAGG	Primerdesign, UK
hTP53	GTGGAGTATTTGGATGACAGAAAC<br>GTAGTTGTAGTGGATGGTGGTAC	Primerdesign, UK
hCCND1	GACCTTCGTTGCCCTCTGT<br>GCGGTAGTAGGACAGGAAGTT	Primerdesign, UK
mCamk2a	ACCACTTCCTTCCACCACTT<br>TGAGATACAGCATTCCATACAAGA	Primerdesign, UK
mPlcb1	TCAAACCCAATCCGATATGTCAAT<br>AGCCTCCTTCTTTACTTCCTCTT	Primerdesign, UK
mDaam1	GGAAGGAGGAGGAGGAACG<br>TCCACTCTCTTCACTGCTCTC	Primerdesign, UK
mPtk7	AATGCTGGAACCCTACACTTTG<br>CCCTGTGGCTCGTTGGAT	Primerdesign, UK
hCAMK2A	AGCAAAGCACTGGGAATTTCT<br>AGGGAAAGAGGGAAAGAGGAAA	Primerdesign, UK

hPLCB1	CTCAGCCCCTTTCTCACTATTTC<br>ACACAGCGACAACCAGACA	Primerdesign, UK
hDAAM1	GAGAAGGAAGAAGAAGAAGAAAGAAG<br>TTTAGAAAGTTGAGGATACATGATAGG	Primerdesign, UK
hPTK7	CGTGGTAGTAGCGAGGTATGA<br>GCGGTTAGTGATGGGAGTCT	Primerdesign, UK
mGapdh	undisclosed	Primerdesign, UK
hGAPDH	undisclosed	Primerdesign, UK
